# Supplementary material for: Modelling the contribution of the hypnozoite reservoir to Plasmodium vivax transmission
Source: eLife. 2014 Nov 18;3:e04692. doi: 10.7554/eLife.04692 (PMC4270097; doi:10.7554/eLife.04692)
Supplement: Supplementary file 1. — Estimated parameters for the within-host relapse. DOI: http://dx.doi.org/10.7554/eLife.04692.018 [file elife04692s001.docx]

**Supplementary file 1**

**Estimated model parameters.**

Prior and posterior medians are presented with 95% credible intervals.

| **parameter** | **description** | **prior median** | **posterior median** |
| --- | --- | --- | --- |
| *Joint parameters (across all regions)* | |  |  |
| *µ* | rate of hypnozoite/hepatocyte death | 1/200 (1/531, 1/104) day^-1^ | 1/425 (1/537, 1/349) day^-1^ |
|  |  |  |  |
| *South America* | | | |
| *N* | number of hypnozoites per infection | 10 (1, 28) | 9.2 (4, 23) |
| *α* | rate of hypnozoite activation | 0.5 (0.025,0.975) day^-1^ | 1/1024 (1/537, 1/349) day^-1^ |
|  |  |  |  |
| *South East Asia* | | | |
| *N* | number of hypnozoites per infection | 10 (1, 28) | 8.5 (4, 32) |
| *α* | rate of hypnozoite activation | 0.5 (0.025,0.975) day^-1^ | 1/332 (1/942, 1/150) day^-1^ |
|  |  |  |  |
| *Melanesia* | | | |
| *N* | number of hypnozoites per infection | 10 (1, 28) | 54 (33, 73) |
| *α* | rate of hypnozoite activation | 0.5 (0.025,0.975) day^-1^ | 1/2440 (1/3915, 1/1501) day^-1^ |
